# Increase of IL-12 following Reconstruction for Total En Bloc Spondylectomy Using Frozen Autografts Treated with Liquid Nitrogen

**DOI:** 10.1371/journal.pone.0064818

**Published:** 2013-05-29

**Authors:** Hideki Murakami, Satoru Demura, Satoshi Kato, Hideji Nishida, Katsuhito Yoshioka, Hiroyuki Hayashi, Kei Inoue, Takashi Ota, Kazuya Shinmura, Noriaki Yokogawa, Xiang Fang, Hiroyuki Tsuchiya

**Affiliations:** Department of Orthopaedic Surgery, Kanazawa University, Kanazawa, Japan; Shanghai Jiao Tong University School of Medicine, China

## Abstract

**Background:**

Total en bloc spondylectomy (TES) is a surgery designed to achieve complete resection of a malignant spinal tumor such as spinal metastasis. Although this procedure decreases the rate of local recurrence, it is questionable whether local control prolongs patient’s survival. We developed a “second-generation TES” that brings about TES enhancing antitumor immunity to prolong patient’s survival. Our purpose is to present a second-generation TES applied tumor-induced cryoimmunology and assess the immunity-enhancing effect after implementing this surgery.

**Methods:**

We performed a retrospective review of prospectively collected data in second-generation TES as carried out in 56 cases. In second-generation TES, instead of harvesting autograft from the ilium or fibula, the resected lamina and vertebral body from TES are frozen using liquid nitrogen and used as grafted bone for spinal reconstruction. In recent 26 of the 56 cases, in addition to the TES procedure, a small amount of the tumor tissue from the resected tumor-bearing vertebra was also placed into liquid nitrogen. This small amount of tumor tissue was then implanted subcutaneously on one side of the axilla. In 52 of 56 cases, measurement of interleukin 12 (IL-12) was performed before surgery and at both 1 and 3 months after surgery.

**Results:**

IL-12 increased after TES surgery in 38 of 52 cases (73.1%). The mean IL-12 relative concentrations at both 1 and 3 months after surgery, as compared to before surgery, were significantly higher (121±89 and 149±111%: P<0.05 and P<0.01).

**Conclusions:**

Our results show that second-generation TES may provide not only a local radical cure but also a systemic immunological enhancement. Further prolonged survival in patients with a malignant spinal tumor is promising by second-generation TES.

## Introduction

Total en bloc spondylectomy (TES) is an excisional surgery designed to achieve complete resection of an aggressive benign spinal tumor or a malignant spinal tumor including spinal metastasis, in addition to providing an adequate tumor margin [1]. This procedure has been reported to decrease the rate of local recurrence since local radical cure is achieved [2], [3]. However, it is questionable whether local control prolongs patient’s survival.

In cryosurgery, antitumor immunity is activated after percutaneous cryoablation of the tumor such as in breast cancer, prostate cancer, renal cell carcinoma, and hepatocellular carcinoma [4], [5]. It has been reported that the metastatic lesions decreased in size or the metastases disappeared because of the cryoimmunological effect after cryosurgery [6]. We applied this tumor-induced cryoimmunology to TES surgery. We have developed a “second-generation TES” that brings about TES enhancing antitumor immunity. In second-generation TES, instead of harvesting autograft from the ilium or fibula, the resected lamina and vertebral body from TES are frozen using liquid nitrogen and used as grafted bone for spinal reconstruction. This provides promise of improving the patient’s life prognosis. Our purpose is to evaluate the immunity-enhancing effect after implementing second-generation TES.

## Patients and Methods

### Ethics Statements

This study was approved by the ethics committee of Kanazawa University. Written informed consent for the surgery and the entry in research was obtained from all 56 patients.

### Study Patients

Since May 2010 we have performed 56 cases of second-generation TES applied tumor-induced cryoimmunology. We performed a retrospective review of prospectively collected data in the 56 cases (29 males and 27 females). The average follow-up period for the 56 cases was 17.7 months (range 6–32 months). The mean age at surgery for these patients was 53.5 years (range 16–73 years).

Of the 56 cases, 49 involved metastatic tumors and 7 involved primary tumors. Of the 49 cases with a metastatic tumor, the primary organ in 13 cases was the kidney, with 8 breast, 8 thyroid, 3 lung, 2 colon, and 13 were other organs; the other 2 cases had primary unknown tumors. Of the 7 cases with a primary tumor, 4 cases were giant cell tumor and the other 3 cases were malignant tumors: osteosarcoma, synovial sarcoma, and pleomorphic carcinoma. The level of TES in the 56 cases was: thoracic spine in 33, thoracolumbar in 7, and lumbar in 16.

Of all 56 cases, 34 cases (60.7%) had other distant metastases before surgery. Fifteen had lung metastases, 2 had liver metastases, and 26 had other bone metastases.

### Surgical Procedure

**Figure 1:**
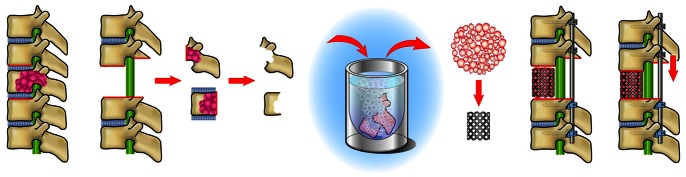
Schema of second-generation TES using tumor-bearing frozen autograft.

Preoperative embolization of bilateral segmental arteries at three levels (i.e., embolization of bilateral segmental arteries of the tumorous and two adjacent, one cephalad and caudad, vertebrae) was performed within 72 hours before operation in all 56 patients. In each of the 56 cases, after en bloc excision of the vertebra (or vertebrae), we used the resected lamina and vertebral body (including the tumor) for bone graft in the anterior spinal reconstruction; we used this bone instead of harvesting autograft from the iliac crest or fibula. To carry this out, we proceeded as follows: After en bloc laminectomy and en bloc corpectomy, the tumor and soft tissue (such as ligament, disc and cartilage) were curetted away from the resected spine. The spine was then placed into liquid nitrogen (Uno Sanso Co., Ltd, Ishikawa, Japan) (−196°C) for 20 minutes. Usually, around 5 minutes is enough for killing tumor cells. However, to freeze the center of the resected spine, we chose 20 minutes just to be safe. The frozen spine was then crushed, and packed into a titanium cage. The cage was placed between the adjacent healthy vertebral bodies. Finally, spinal shortening was carried out in order to stabilize the cage (Figure 1).

In addition to the procedure described above, in the most recent 26 cases, a small amount of the tumor tissue from the resected tumor-bearing vertebra was also placed into liquid nitrogen for 20 minutes. This small amount of tumor tissue was then implanted subcutaneously on one side of the axilla at the end of this surgery.

### Evaluation of Antitumor Immunity Enhancement

Blood samples were collected from the patients before surgery and at 1 and 3 months after surgery for the measurement of interleukin 12 (IL-12) in order to assess the immunity-enhancing effect. These analyses were available in 52 of the 56 cases. Four patients were not available because their blood samples could not be collected at an appropriate time. We evaluated the increase-decrease rate of IL-12. Moreover, the increase-decrease rate of IL-12 in the 26 patients who had received the small amount of tumor tissue implanted subcutaneously, was compared with that in the 26 patients without implantation.

Whole-body computed tomography (CT) or/and spinal MRI were taken 1, 3, 6, 12, and 24 months after surgery in all 56 cases to evaluate local recurrence and distant metastases.

### Statistical Analysis

Continuous variables were expressed as median±quartile deviation. Statistical differences were calculated by the Wilcoxon signed-rank test and the Mann-Whitney *U* test. Statistical significance was set at p<0.05. SPSS for Windows (19.0; SPSS Inc., Chicago, IL, USA) was used to perform the statistical analyses.

## Results

**Figure 2:**
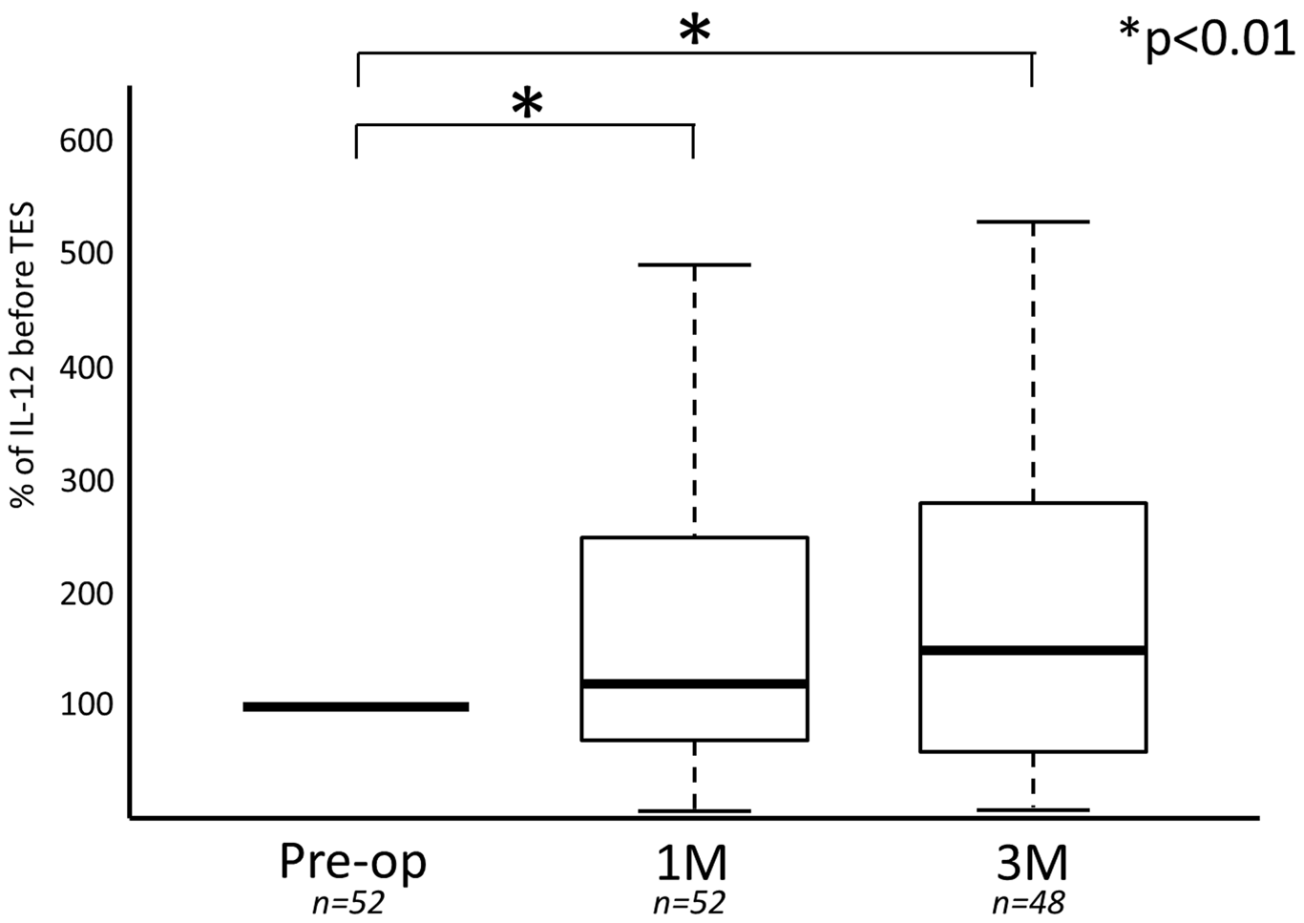
Serial changes of IL-12 concentrations at 1 month and 3 months after TES were compared with the concentration before TES.

**Figure 3:**
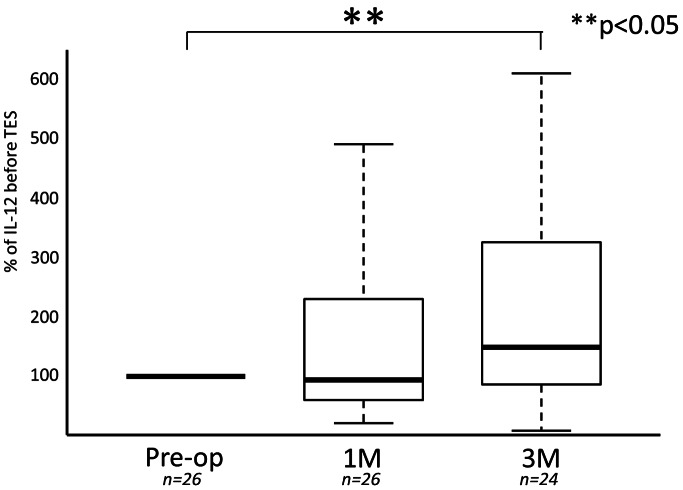
Serial changes of IL-12 concentrations at 1 month and 3 months after TES were compared with the concentration before TES in the 26 cases without tumor tissue implantation.

**Figure 4:**
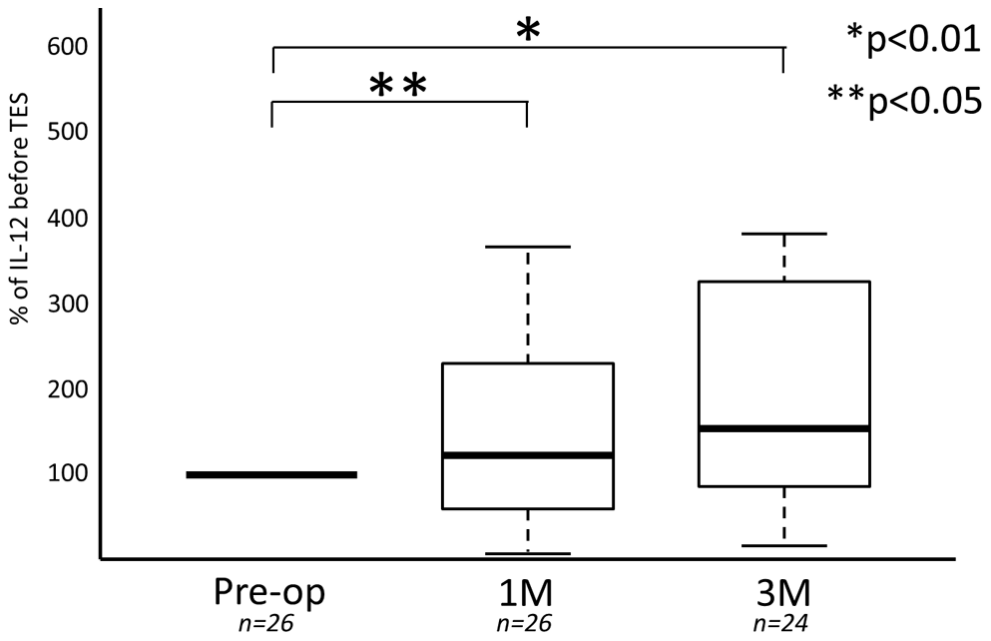
Serial changes of IL-12 concentrations at 1 month and 3 months after TES were compared with the concentration before TES in the 26 cases with tumor tissue implantation.

**Figure 5:**
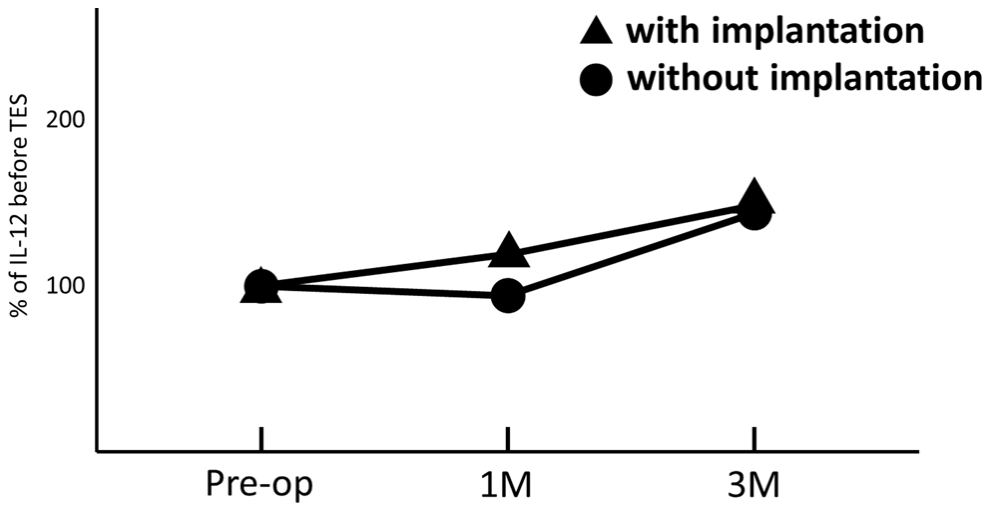
Longitudinal comparison between without implantation and with implantation.

IL-12 increased after surgery in 38 of 52 cases (73.1%). Evaluating all 52 cases the mean IL-12 relative concentrations at both 1 and 3 months after surgery, as compared to before surgery, were significantly higher (121±89 and 149±111%: P<0.05 and P<0.01) (Figure 2). In the series of 26 cases without subcutaneous implantation of the tumor tissue, the mean IL-12 relative concentrations at both 1 and 3 months after surgery, as compared to before surgery, were higher (93±90 and 146±120%: P>0.05 and P<0.05) (Table 1). The increase of the mean IL-12 relative concentrations at 3 months was significant (Figure 3). Blood samples were not collected from 2 of 26 patients at 3 months after surgery because they could not visit our clinic for geographical reasons. In series of 26 cases with subcutaneous implantation of the tumor tissue, the mean IL-12 relative concentrations at both 1 and 3 months after surgery, as compared to before surgery, were significantly higher (123±74 and 152±89%: P<0.05) (Table 2) (Figure 4). Blood samples were not collected from 2 of 26 patients at 3 months after surgery because they could not visit our clinic for geographical reasons. The percentage increase of IL-12 in patients with subcutaneous implantation of tumor tissue was higher than that in patients without implantation at both 1 and 3 months after surgery (Figure 5). However, these increases were not significant.

**Table 1:** Patients without frozen tumor tissue implantation (n = 26).

PatientNumber	Age	Sex	Tumor	Excised Vertebra	IL-12(pre-op)	IL-12(1 M)	IL-12(3 M)	Frankel(pre-op)	Frankel(follow-up)
1	71	M	RCC met	C7-T2	2.7	54.1	16.5	C	E
2	67	M	RCC met	T11-L1	43.0	23.5	124.0	C	E
3	73	M	Prostate ca met	C7-T1	5.4	71.0	12.3	C	D
4	62	F	RCC met	L1-3	3.6	23.8	15.8	C	C
5	69	F	Thyroid ca met	T3-5	117.0	24.0	35.8	E	E
6	61	M	RCC met	T3	335.0	559.0		E	E
7	19	F	Osteosarcoma	T2-5	2.8	13.7	97.5	A	A
8	52	M	Pleomorphic carcinoma	T1-4	17.7	8.3	28.9	C	A
9	67	F	Thyroid ca met	C7-T2	7.9	6.0	10.5	C	C
10	39	M	Thyroid ca met	T4	29.7	52.3	137.0	E	E
11	57	F	Thyroid ca met	T5-6	13.4	34.5	20.0	E	E
12	56	F	Breast ca met	T9	113.0	28.8	28.4	E	E
13	39	M	Thyroid ca met	L4	52.3	37.9	24.9	E	E
14	41	F	Synovial sarcoma	T8-9	8.6	8.1	12.3	E	E
15	54	F	Chondrosarcoma	T7-9	55.2	24.1	22.1	E	E
16	69	M	Leiomyosarcoma met	T10-11	27.9	78.0	64.8	E	E
17	67	F	Thyroid ca met	T8	70.9	103.0	61.8	E	E
18	49	F	GCT	L4-5	9.9	9.1	119.0	D	D
19	42	M	RCC met	T7-8	25.2	12.9	29.9	E	E
20	64	M	GIST met	T5	239.0	207.0	24.9	E	E
21	52	M	Epipharyngeal ca met	T4	18.1	108.0	95.4	B	C
22	70	M	RCC met	L3	93.6	74.1	77.6	E	E
23	62	M	RCC met	T12	16.8	30.8	35.7	E	E
24	25	F	GCT	L4	13.9	21.3	18.3	E	D
25	58	M	Lung ca met	T5	35.1	11.1	35.1	E	E
26	59	F	Primary unknown ca met	T6	39.3	27.2		E	E

Abbreviations: RCC, renal cell carcinoma; ca, cancer; met, metastasis; GCT, giant cell tumor; GIST, Gastrointestinal mesenchymal tumor.

**Table 2:** Patients with frozen tumor tissue implantation (n = 26).

PatientNumber	Age	Sex	Tumor	ExcisedVertebra	IL-12(pre-op)	IL-12(1 M)	IL-12(3 M)	Frankel(pre-op)	Frankel(follow-up)
27	46	F	Breast ca met	T4-6	12.5	130.0	227.0	C	D
28	61	M	Gastric ca met	L4	3.3	5.1	4.9	E	D
29	43	M	RCC met	L4	32.7	39.9	21.7	E	E
30	53	F	Uterus ca met	T10-11	8.5	10.7	14.0	E	E
31	73	F	Thyroid ca met	T8-9	51.3	28.2	39.5	E	E
32	63	F	Thyroid ca met	T4-6	7.2	78.4	27.3	C	E
33	62	F	Breast ca met	T1-3	124.0	99.6	39.8	C	E
34	24	F	Leiomyosarcoma met	T7-8	8.2	20.2	372.0	E	E
35	49	M	Colon ca met	T10	11.4	6.4	3.8	E	E
36	61	M	Esophagus ca met	T11	14.6	12.1	19.9	E	E
37	58	M	Colon ca met	T12	17.9	22.2	152.0	E	E
38	68	M	RCC met	T7	54.7	65.0	28.7	E	E
39	50	M	Primary unknown ca met	L5	9.9	10.6	21.2	E	D
40	50	M	Renal pelvic ca met	L1	14.9	25.4	23.1	D	D
41	46	M	RCC met	T1-3	60.4	53.1	113.0	E	E
42	51	F	Urachal ca met	L3	20.5	8.4	9.8	E	E
43	59	M	RCC ca met	T8-10	48.8	179.0	90.9	E	E
44	53	M	Lung ca met	T2-4	2.4	15.5	29.5	E	E
45	44	F	Breast ca met	L1	24.8	12.1	12.1	E	E
46	42	F	Breast ca met	T12-L2	9.3	56.6	25.3	E	E
47	62	M	RCC met	T2-4	28.3	26.3		A	A
48	66	F	Breast ca met	T11-12	10.0	34.7	16.3	E	E
49	65	F	RCC met	L1-2	148.0	8.2	45.6	E	E
50	66	F	Breast ca met	T10	60.2	116.0	8.7	E	E
51	45	M	Chondrosarcoma met	T10	58.8	100.0		E	E
52	26	F	Angiosarcoma met	T2-4	7.6	7.8	5.9	E	E

Abbreviations: RCC, renal cell carcinoma; ca, cancer; met, metastasis.

At final follow-up, 10 of the 56 patients had died due to progression of metastases (mean 10.9 months after TES), 12 remained free from disease and 34 patients were alive with disease. Of the 46 patients that were still alive at final follow-up, there has not been growth of metastatic lesions or emergence of new metastases in 22 cases from the results of whole-body CT. Surprisingly, in one case of a patient with breast cancer metastases (patient 27 in Table 2), lymph node metastasis clearly decreased in size after second-generation TES; this patient had received no other treatments (Case presentation, see below). In 2 cases of thyroid cancer metastases (patient 10 in Table 1 and patient 31 in Table 2), the level of tumor marker (serum thyroglobulin) decreased in postoperative course without any other treatments.

There were 3 cases of local recurrence after this surgery. In the 3 cases, one with metastasis of epipharyngeal cancer (patient 21 in Table 1), one with gastric cancer (patient 28 in Table 2), and the other with colon cancer (patient 35 in Table 2), recurrence occurred from tissues around the spinal column. Recurrence was not detected in the grafted bone inside the cage. There were no failures of the cage or adverse effects from the use of frozen autograft in all cases. Neurological symptoms (Frankel grade) of 56 cases before surgery and at follow-up are presented in Table 1 and 2.

## Case Presentation

### Written Consent was Obtained for the Publication of this Case

**Figure 6:**
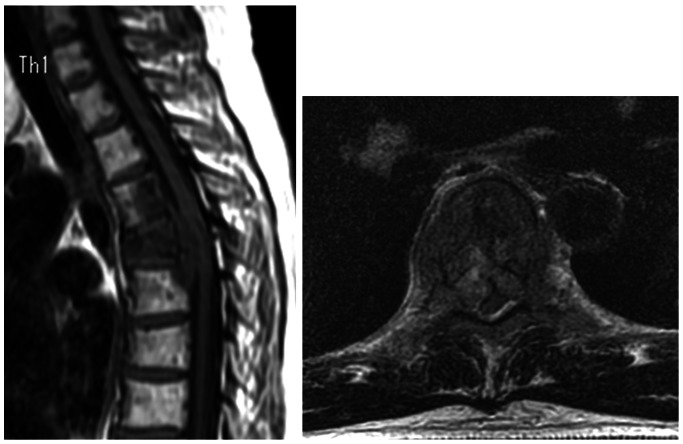
Preoperative MRI. This patient had metastasis invading into the spinal canal at the T5 level.

**Figure 7:**
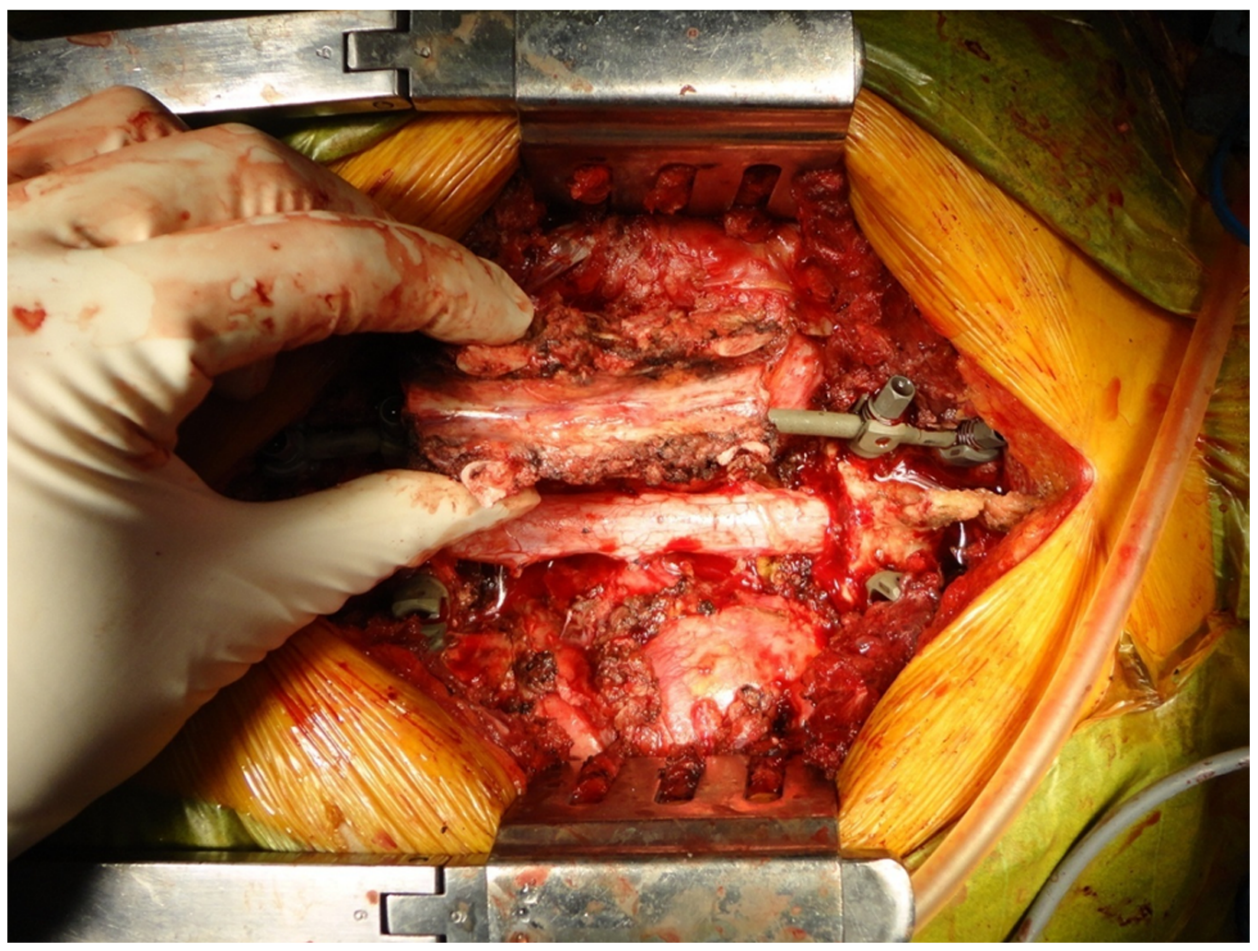
Operative photograph after en bloc corpectomy. En bloc corpectomy of T4-6 was performed.

**Figure 8:**
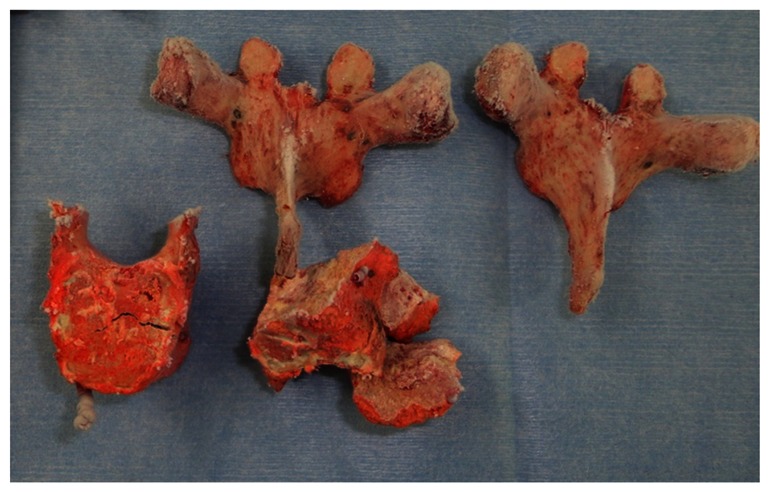
Frozen tumor-bearing vertebrae.

**Figure 9:**
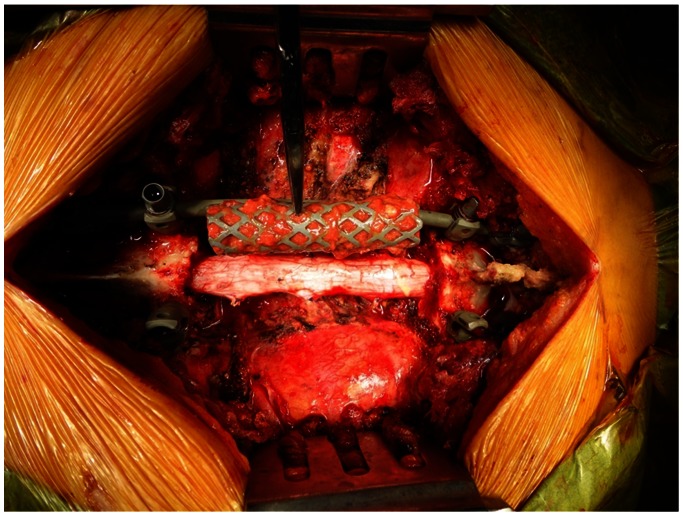
Operative photograph before inserting a cage containing frozen autograft.

A 46 year-old female with metastasis of breast cancer at T4-6 was referred to our hospital. Five years before, she had received a mastectomy based on the diagnosis of right breast cancer (T2N1M0 stage IIIB). After surgery she underwent postoperative chemotherapy. Three years after the mastectomy (2 years ago) multiple bone metastases and liver metastasis were detected, and chemotherapy and radiation were performed. Six months ago, a new lesion of liver metastasis and axillary lymph node metastasis were detected and hormone therapy was started. When she was admitted to our hospital, she had multiple liver metastases, multiple bone metastases (rib, lumbar spine, sacrum, and pubis), and axillary lymph node metastasis. Metastasis of T5 had invaded into adjacent vertebrae above and below (T4 and T6) and the spinal cord was severely compressed (Figure 6). She had upper back pain with paralysis of her bilateral lower limbs. Her Frankel grade was C and she could not stand without supports. TES of T4 to T6 was performed (Figure 7). The excised en bloc laminae and en bloc vertebral bodies of T4 to T6 were placed into liquid nitrogen for 20 minutes. Then, the frozen laminae and vertebral bodies (Figure 8) were crushed and packed into a titanium cage. The cage was placed between T3 and T7 for spinal anterior reconstruction (Figure 9). At the end of surgery, a small amount of the tumor tissue from the resected vertebrae (T4 to T6) was also placed into liquid nitrogen for 20 minutes. The frozen tumor tissue was then implanted under the skin of right axilla.

**Figure 10:**
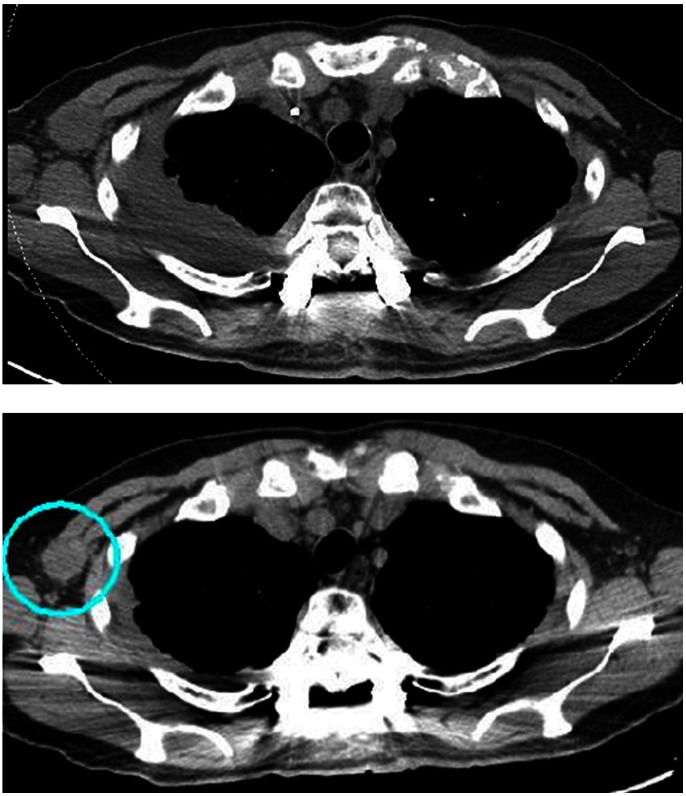
Postoperative computed tomography of the lung (Top; immediately after TES, Bottom; 1 month after TES). Right axillary lymph node metastasis is reduced. A circle shows reduced lymph node of right axilla.

**Figure 11:**
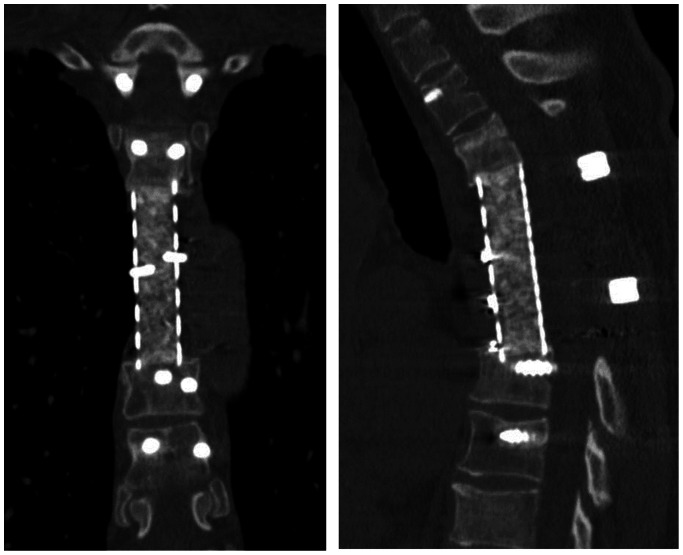
Postoperative computed tomography of the thoracic spine (Left; frontal view, Right; lateral view) one year after TES. Bony fusion can be seen inside the cage.

At one month after surgery, the axillary lymph node metastasis decreased in size without any other treatments (Figure 10). Her muscle strength of the bilateral lower extremities gradually improved after TES. Her Frankel grade became D and she could walk with a cane at follow-up (18 months after TES). The lymph node metastasis was stable at follow-up. Some of the multiple liver and bone metastases had reduced in size and some had increased in size. Before TES the prognosis given by the specialists in breast cancer was 6 months to 1 year (maximum 1 year); she is still alive with disease 18 months after TES (Figure 11).

## Discussion

Although we use tumor-bearing vertebra as grafted bone, the tumor cells are completely killed by being placed into liquid nitrogen for 20 minutes. Tsuchiya et al. have already reported on bone reconstruction for malignant tumors in the extremities and pelvis being treated with tumor-bearing autograft frozen by being placed in liquid nitrogen [7]. Their results give some proof that reconstruction using frozen tumor-bearing bone is a safe and effective method. Moreover, local recurrences from tumor-bearing autograft have not been reported at all. In our series, no recurrences were detected in the grafted bone inside the cage.

Promising developments building upon standard cancer therapies, such as chemotherapy, radiotherapy and surgery, have dramatically extended patient survival for malignant tumors. In addition, immunologic therapies such as exogenous cytokines, dendritic cell therapy and peptide vaccines have yielded promising initial results in the clinical setting. Cryoimmunology has also shown promising results in some malignant tumors after cryosurgery and is expected to influence the next generation of tumor immunotherapy [4]–[6]. We have described in this study the induction of a systemic antitumor immune response following second-generation TES using frozen tumor-bearing vertebra and frozen tumor tissue treated with liquid nitrogen. This cryoimmunological response via dendritic cells in second-generation TES is expected to herald a new surgical concept to promote tumor-specific immune responses that subsequently enhance systemic immune responses.

In this second-generation TES, tumor cells are completely destroyed by liquid nitrogen. On the other hand, tumor antigens are preserved. The tumor antigens released from the necrotic tumor cells in tumor-bearing grafted bone inside the cage activate the tumor-specific immune response. Released tumor antigens are ingested by dendritic cells. The dendritic cells migrate to local lymph nodes. In the lymph nodes, the dendritic cells act as antigen-presenting cells. Since dendritic cells mainly exist subcutaneously, small amount of frozen tumor tissue was implanted subcutaneously in our surgical procedure. Moreover, since dendritic cells work in lymph nodes, frozen tumor tissue was placed near axillary lymph nodes. Previous reports have suggested immune system activation brought about by cryosurgery [4], [5], [8], [9]. Nishida et al. reported that re-implantation of tumor tissue frozen using liquid nitrogen induces antitumor activity against murine osteosarcoma [10] and the serum levels of INF-γ and IL-12 increased after treatment [11]. Moreover, they reported on a patient with metastases from renal cell carcinoma involving the lungs and bone (femur). The lung metastases diminished after femoral reconstruction using the resected femur treated by liquid nitrogen for the bone metastasis [12]. The antitumor effect by the systemic immune reaction in second-generation TES could inhibit metastatic tumor growth and local recurrence.

IL-12, also known as a natural killer cell stimulatory factor or a cytotoxic lymphocyte maturation factor, is a pleiotropic cytokine. IL-12 is produced by dendritic cells that are critical professional antigen-presenting cells, and IL-12 has multiple effects on T cells and natural killer cells. These include proliferation and activation of natural killer cells and cytotoxic T lymphocytes directly, and induction of type 1 helper T cells which activate cytotoxic T lymphocytes. IL-12 relative concentrations showed significant increases at both 1 and 3 months after second-generation TES. This means that the possible occurrence of a systemic antitumor immune response was induced by second-generation TES. Moreover, further enhancement of antitumor immunity by the implantation of tumor tissue (as under the skin of the axilla in our patients) can be reasonably expected. To prove antitumor immune response by second-generation TES more securely, it is better to evaluate other immune parameters besides IL-12. We are newly collecting clinical data of not only IL-12 but also interferon-gamma (IFN-γ), type 1 helper T-cell/type 2 helper T-cell (Th1/Th2) ratio, and regulatory T-cell (Treg).

Second-generation TES using frozen autograft inside a cage as presented here affords three other benefits: 1) no pain at the bone harvest site; 2) shortening of the operation time; and 3) decreased blood loss. These three benefits hasten recovery from TES surgery. In addition, biological healing of the frozen autograft inside the cage would seem to offer promise since the bone is both osteoinductive and osteoconductive [7]. Proteins such as bone morphogenic proteins are preserved in frozen autograft. Tanzawa et al. have reported biological healing of frozen bone histopathologically [13].

Our results show that antitumor immunity can be enhanced after second-generation TES. However, we have not been able to compare patients’ survival in our novel TES technique with the previous TES technique, since sample size was small and follow-up period was still short in this study. Moreover, we have performed second-generation TES even for patients with advanced stage of cancer. In this study, 34 (60.7%) of 56 cases have already had other distant metastases such as lung, liver and so on before surgery. From our results, it is difficult to present prolonged patients’ survival by immune response after second-generation TES. Although we have still to collect clinical data, second-generation TES as presented here, may provide not only a local radical cure but also a systemic immunological enhancement. Further prolonged survival may be enhanced by the antitumor effect against disseminated tumor cells throughout the body. In the past, the indication for TES was limited. The indication has been for solitary spinal metastasis without distant metastasis to vital organs. However, we recommend that the indication for TES be extended. Based on the procedures and results presented here, existence of metastases to vital organs such as lung or liver is not sufficient reason for not implementing TES.
